# Cytokine serum levels during post-transplant adverse events in 61 pediatric patients after hematopoietic stem cell transplantation

**DOI:** 10.1186/s12885-015-1616-z

**Published:** 2015-08-28

**Authors:** Michaela Döring, Karin Melanie Cabanillas Stanchi, Markus Mezger, Annika Erbacher, Judith Feucht, Matthias Pfeiffer, Peter Lang, Rupert Handgretinger, Ingo Müller

**Affiliations:** 1 Department I – General Paediatrics, Hematology/Oncology, University Hospital Tuebingen, Children’s Hospital, Hoppe-Seyler-Str. 1, 72076 Tuebingen, Germany; 2 Department of Paediatric Hematology and Oncology, University Hospital Hamburg-Eppendorf, Center for Obstetrics and Paediatrics, Martinistraße 52, 20246 Hamburg, Germany

## Abstract

**Background:**

Veno-occlusive disease, Graft-versus-Host disease, invasive or localized bacterial, viral and fungal infections are known as adverse events after hematopoietic stem cell transplantation representing the major cause for morbidity and mortality. Detection and differentiation of these adverse events are based on clinical symptoms and routine measurements of laboratory parameters.

**Methods:**

To identify the role of cytokines as a possible complication-marker for adverse events, 61 consecutive pediatric patients with a median age of 7.0 years who underwent hematopoietic stem cell transplantation were enrolled in this single-center retrospective study. Interleukin-1 beta (IL-1β), soluble interleukin-2 receptor (sIL-2R), interleukin-6 (IL-6), interleukin-8 (IL-8), interleukin-10 (IL-10) and tumor necrosis factor-α serum (TNF-α) levels were regularly assessed after transplantation and during transplantation related adverse events.

**Results:**

Veno-occlusive disease was accompanied by a significant increase in levels of IL-6, IL-8 and TNF-α.Graft-versus-Host disease was associated with a significant increase of IL-10, sIL-2R, IL-6 and TNF-α, depending on the respective stage or grade. Cytokine IL-6 enabled a significant differentiation between sepsis and fungemia, sepsis and viremia, and sepsis and bacteremia. Moreover, cytokine IL-8 enabled a significant differentiation between sepsis and viremia, sepsis and bacteremia, and bacteremia and viremia whereas IL-10 made a distinction between sepsis and viremia possible.

**Conclusion:**

The data demonstrate that proinflammatory cytokines might be putative indicators for early detection and differentiation of post-transplant adverse events and may allow prompt and adequate clinical intervention. Prospective clinical trials are needed to evaluate these findings.

## Background

Post-transplant adverse events such as sepsis, bacterial, viral or fungal infections, acute Graft-versus-Host disease (GvHD) and veno-occlusive disease (VOD) are major causes of morbidity and mortality after hematopoietic stem cell transplantation (HSCT) [1–6]. Numerous reports have demonstrated that certain cytokines are released during the conditioning and post-transplant periods [7–12]. Interleukin 8 (IL-8) is known to increase drastically one to four days after the diagnosis of a severe VOD while soluble interleukin-2 receptor sIL-2R (sIL-2R) seems to increase significantly during VOD [13, 14]. This increase was reported to be significantly higher than in patients with GvHD grade II or III during the post-transplant period. Patients with VOD or GvHD grade II or III experience an increase of the inflammatory cytokines interleukin 6 (IL-6) and tumor necrosis factor-α (TNF-α). Patients with severe acute GvHD grade III or IV after HSCT show a significant increase in interleukin 10 (IL-10) levels between the aplastic phase and the leukocyte recovery phase after transplantation in comparison to patients that do not develop GvHD [15]. In the first 15 weeks of the post-transplantation period, serum levels of sIL-2R and IL-10 are significantly higher in transplanted patients that develop GvHD than in patients without GvHD [16]. Patients with higher levels of TNF-α and IL-10 at two weeks after HSCT develop moderate-to-severe GvHD in the post-transplant period in comparison to patients with relatively lower TNF-α and IL-10 levels which can be correlated with a lower GvHD grade [17]. An increase in IL-6 and IL-10 levels can be observed during acute GvHD grade II and more in the early post-transplant period, while the levels of TNF-α and IL-8 remain unchanged [10]. Other studies have reported an increase in TNF-α level at the onset of GvHD [18–20].

IL-6 plays a decisive role in the trans-signaling pathogenesis of sepsis [21]. It could be shown that IL-6 and IL-8 are reliable indicators that enable the differentiation of pediatric oncology patients with short duration of fever episodes from patients with severe infection or even blood culture positive sepsis [22]. IL-8 was shown to be a highly sensitive predictor for pediatric oncology patients at low risk for bacteremia [23] while IL-10 was shown to correlate with bacteremia and sepsis [24]. In 14 HSCT recipients with a human herpes virus 6 (HHV-6) reactivation after HSCT, IL-6 and TNF-α levels were significantly higher than in recipients without an HHV-6 viremia [9]. Further, it could be shown that renal transplant patients that suffer from post-transplant cytomegalovirus (CMV) viremia develop increased sIL-2R, IL-6, and IL-10 cytokine levels [25]. Serum levels of IL-8, IL-6, IL-10 and C-reactive protein (CRP) could be used as differentiation markers for high and low risk pediatric oncology patients with neutropenia [26] whereas in adult oncology patients, serum concentrations of CRP, IL-6, IL-8 and sIL-2R were elevated in the afebrile neutropenic period [27].

Taken together, the presented results display the crucial role of cytokines in these immunologic phenomena but still, sufficient knowledge about cytokine pattern is not available yet for early identification and differentiation of the various types of adverse events such as localized viral infections. It is currently not possible to identify and distinguish a VOD from an acute liver GvHD, a bacteremia from a viremia or fungemia, or diarrhea caused by a localized viral infection in feces from an intestinal GvHD. In order to provide insight into these issues the present study addresses the analysis of interleukin 1β (IL-1β), sIL-2R, IL-6, IL-8, IL-10, and TNF-α serum levels in regular intervals after allogeneic and autologous HSCT in pediatric patients. The data were analyzed with respect to the patient’s clinical presentation.

The priority objective of the present study was to analyze whether early identification of major post-transplant related adverse events in pediatric patients with hemato-oncological malignancies and non-malignancies after allogeneic and autologous HSCT is possible through the examination of cytokine levels.

## Methods

### Ethics

This analysis was conducted in accordance with the Declaration of Helsinki and performed under the waiver for retrospective anonymized studies in accordance with the Independent Ethics Committee (IEC) of the Eberhard-Karls-University Tuebingen. Written informed consent was obtained by the patients or their legal representatives.

### Survey design and patient characteristics

This retrospective single-center investigation comprises a longitudinal analysis of cytokine levels IL-1β, sIL-2R, IL-6, IL-8, IL-10, and TNF-α of consecutive pediatric patients before, during and after allogeneic (*n* = 59) and autologous (*n* = 2) HSCT. The analysis was a single cohort, with baseline samples from each patient, which was subsequently divided into a group of patients without complications during therapy and patients with one of several defined complications. The observation period was defined as the period from the day before start of the conditioning regimen until the date of discharge after HSCT. The patient group consisted of 61 pediatric patients and young adults (36 males, 25 females) with a median age of 7.0 years (range 0.5 – 26 years) undergoing HSCT for hemato-oncological malignancies and inborn errors of metabolism. Patients received transplants from mismatched family donors (MMFD, *n* = 38), matched unrelated donors (MUD, *n* = 16), HLA-identical siblings (*n* = 5) or patients who underwent autologous transplantation (*n* = 2). All autologous and allogeneic transplant recipients received standard prophylaxis including antimycotics, virostatics and metronidazole. On day four after HSCT, all allogeneic transplanted patients received granulocyte colony-stimulating factor (G-CSF) at a dosage of 5 μg per kg body weight and day (mg/kg BW/d) until leukocytes stabilized (>1000/μl) and neutrophils reached levels of >500/μl. GvHD prophylaxis was applied depending on the type of transplantation intravenously with cyclosporine A (CsA), mycophenolate mofetil, anti-thymocyte globulin (ATG), methotrexate or muromonab-CD3.

Thirteen of the 61 patients did not suffer from post-transplant complications such as VOD, GvHD, sepsis, invasive or localized bacterial, viral or fungal infection. Patient characteristics are summarized in Table 1.

**Table 1:** Patient characteristics

	Patients without		Patients with	
	post-transplant adverse events			
	*n* = 13		*n* = 48	
	No. [%]		No. [%]	
Sex				
Male	7	(53.8)	29	(60.4)
Female	6	(46.2)	19	(39.6)
Age group				
<6 y	6	(46.2)	15	(31.3)
6–12 y	3	(23.1)	18	(37.5)
13–26 y	4	(30.8)	15	(31.3)
Donor				
MUD	3	(23.1)	13	(27.1)
MMFD	7	(53.8)	31	(64.6)
MFD	1	(7.7)	4	(8.3)
Autologous	2	(15.4)	0	(0.0)
Primary diagnosis				
ALL	1	(7.7)	11	(22.9)
ALL relapse	0	(0.0)	11	(22.9)
AML	0	(0.0)	3	(6.3)
AML relapse	1	(7.7)	4	(8.3)
CML	1	(7.7)	2	(4.2)
JMML	1	(7.7)	1	(2.1)
MDS	0	(0.0)	4	(8.3)
T-NHL	0	(0.0)	1	(2.1)
Solid tumors	3	(23.1)	3	(6.3)
Aplastic anemia	0	(0.0)	1	(2.1)
Neurometabolic disease	1	(7.7)	3	(6.3)
Immunologic disease	2	(15.4)	3	(6.3)
Autoimmune disease	2	(15.4)	0	(0.0)
Chédiak-Higashi syndrome	1	(7.7)	0	(0.0)
Kostmann disease	0	(0.0)	1	(2.1)
Neurologic disease	0	(0.0)	0	(0.0)
Radiation				
TLI	0	(0.0)	3	(6.3)
TBI	1	(7.7)	7	(14.6)
GvHD prophylaxis				
CsA + MTX	3	(23.1)	11	(22.9)
CsA	1	(7.7)	6	(12.5)
Acute GvHD				
Grade I	0	(0.0)	11	(22.9)
Grade II	0	(0.0)	9	(18.8)
Grade III	0	(0.0)	3	(6.3)
Grade IV	0	(0.0)	1	(2.1)

*Abbreviations*: *ALL* acute lymphoblastic leukemia, *AML* acute myeloid leukemia, *CML* chronic myeloid leukemia, *CsA* cyclosporine A, *GvHD* graft-versus-host disease, *JMML* juvenile myelomonocytic leukemia, *MDS* myelodysplastic syndromes, *MFD* matched family donor, *MMFD* mismatched family donor, *MTX* methotrexate, *MUD* matched unrelated donor, *TBI* total body irradiation, *TLI* total lymphoid irradiation, *T-NHL* T cell non-Hodgkin’s lymphoma, *y* year(s)

### Criteria for the assessment of post-transplant adverse events

Diagnosis of VOD was made according to the Seattle or Baltimore clinical criteria [28–30]. No liver biopsy or analysis of plasminogen activator inhibitor-1 (PAI-1) level was performed in patients diagnosed with VOD. Clinical diagnosis of acute GvHD followed the criteria of Glucksberg and colleagues [31]. Sepsis was evaluated according to the criteria of the International Sepsis Consensus Conference on Paediatric Critical Care 2005 [32]. Bacteremia was diagnosed with at least one positive blood culture. Viremia was defined as a positive polymerase chain reaction analysis resulting from blood for CMV, adenovirus (ADV), HHV-6, Epstein-Barr virus (EBV), varicella zoster virus (VZV), human herpes simplex virus (HSV) and Parvovirus B19. A local bacterial and viral infection, i.e., a non-invasive bacterial, viral or fungal infection in the blood was defined as a positive microbiological or virological test of infection in the throat, urine or feces. Proven or probable invasive fungal infections were defined in accordance with the definitions for invasive fungal diseases presented by the Invasive Fungal Infections Cooperative Group of the European Organization for Research and Treatment of Cancer and the National Institute of Allergy and Infectious Diseases Mycoses Study Group (EORTC/MSG) [33].

### Blood sampling and laboratory analyses

In 61 patients, the levels of the cytokines IL-1ß, sIL-2R, IL-6, IL-8, IL-10 and TNF-α were measured as a part of the routine blood analyses on the morning before start of conditioning, twice during the conditioning period and after HSCT, as well as two times per week up to the time of clinical discharge. The blood samples were taken between 6:00 a.m. and 8:00 a.m.

IL-1ß was measured using an enzyme linked immunosorbent assay (ELISA, R&D Systems, Wiesbaden, Germany). The levels of sIL-2R, IL-6, IL-8, IL-10 and TNF-α were measured by chemoluminescent immunoassays (Immulite, Siemens Healthcare, Erlangen, Germany). The reference values were as followed: <0.5 pg/ml for IL-1ß, <1000 U/ml for s-IL-2R, <5.0 pg/ml for IL-6, <70 pg/ml for IL-8, <10 pg/ml for IL-10 and <8 pg/ml for TNF-α.

### Statistical analysis

All 61 pediatric and adolescent patients were included in the statistical analyses. The analyzed cytokine levels were measured before the start of treatment with the conditioning of all 61 pediatric patients. The time designated before post-transplant adverse event (=baseline) was defined as the time of the last cytokine level measurement before occurrence of any transplant-related adverse event. The time called “post-transplant adverse event”, referred to the first measurement of cytokine levels at the beginning of the first clinical symptoms or laboratory chemical changes, which were related to observed post-transplant adverse events. The cytokine data are presented as median values and range, or means + standard deviation (SD). Non-parametric statistical tests were applied because of frequent non-normality of data sets (tested by the Shapiro-Wilk normality test), or small or unequal sample sizes. The Wilcoxon matched pairs signed rank test was applied for statistical comparisons of the cytokine levels between “before post-transplant adverse event”, and “post-transplant adverse event“. For the comparisons between MUD versus MFD, MUD versus MMFD and total body irradiation (TBI) versus non-TBI, the Mann–Whitney test was used for these unpaired data. The presented values for the group without complications were taken from 13 of the 61 pediatric patients at the point in time “before treatment”. P values of p ≤ 0.05 (*), p ≤ 0.01 (**) and p ≤ 0.001 (***) were defined as statistically significant. The Bonferroni procedure was applied for the correction of multiple testing. The statistical analysis was performed with the statistical program XLStat2010 (AddinSoft, Paris, France). GraphPad Prism® Version 5.04 for Windows (GraphPad Software Inc., La Jolla, CA, USA) was used for creating graphics.

## Results

This retrospective investigation analyzed the role of the cytokines IL1-β, sIL-2R, IL-6, IL-8, IL-10 and TNF-α as potential markers for major post-transplant adverse events including VOD, skin and intestinal GvHD, sepsis as well as bacterial, viral and fungal infections in 61 pediatric patients.

The median observation period was 74 days (range 28–245 days) and included the time of measurement directly before the start of conditioning until the day of clinical discharge.

### Patient group without complications

The group without complications included in this analysis consisted of 13 of the 61 pediatric patients with a median age of 7 years (range 11 months to 18 years). 4 (30.8 %) of the 13 patients had leukemia, 3 (23.1 %) had a solid tumor, 2 (15.4 %) had an immunologic disease, 2 (15.4 %) had an autoimmune disease, 1 (7.7 %) had a neurometabolic disease and 1 (7.7 %) had Chédiak-Higashi-syndrome (Table 1). These patients experienced none of the evaluated adverse events like VOD, acute GvHD, invasive or localized fungal, viral or bacterial infection during the conditioning and the observed post-transplant period. Levels of the cytokines IL-1ß, sIL-2R, IL-6, IL-8, IL-10 and TNF-α, were measured in this cohort twice a week. During the conditioning and post-transplant period, the median level of TNF- α (median 5.8 pg/ml, range 4.0 – 10.0 pg/ml) was elevated (>8 pg/ml) in only 4 of 13 patients. The levels of IL-1ß (median 0.1 pg/ml, range 0.1 – 0.4 pg/ml), sIL-2R (median 585 U/ml, range 296–869 U/ml), IL-6 (median 2.0 pg/ml, range 2.0 – 5.0 pg/ml), IL-8 (median 14.0 pg/ml, range 5.0 – 56 pg/ml), and IL-10 (median 3.1 pg/ml, range 1.0 – 6.9 pg/ml) were within the normal range during the observation period.

### Cytokines and stem cell transplantation

The analysis of the cytokine level in the different types of stem cell transplantation and conditioning regimen occurred at median on day +2 (range +1 to +4) after HSCT. The comparison of patients with versus without TBI did not reveal anystatistically significant difference in any of the cytokines analyzed (IL-1β: *P* = 1.0; sIL-2R: *P* = 0.228, IL-6: *P* = 0.912; IL-8: *P* = 0.645; IL-10: *P* = 0.868; TNF-α: *P* = 0.433). As well, comparison of cytokine levels between MUD and MMFD showed no significant difference (IL-1β: *P* = 0.123; sIL-2R: *P* = 0.588, IL-6: *P* = 0.494; IL-8: *P* = 0.695; IL-10: *P* = 0.793; TNF-α: *P* = 0.426). In contrast to this, the comparison of MUD and MFD showed significant differences for cytokines IL-1β (mean 0.134 ± 0.058 pg/ml versus 0.624 ± 0.184 pg/ml, respectively; *P* = 0.0019), sIL-2R (mean 1431 ± 1076 U/ml versus 550 ± 165 U/ml, respectively; *P* = 0.0185) and IL-8 (mean 46.3 ± 37.6 pg/ml versus 16.0 ± 11.7 pg/ml, respectively; *P* = 0.023). Levels of IL-6 (*P* = 0.067), IL-10 (*P* = 0.221) and TNF-α were not significantly different in these two groups.

### Transplant-related adverse events

#### Veno-occlusive disease

In 5 (8.2 %) of 61 patients, VOD was diagnosed according to the clinical and laboratory criteria. The first clinical symptoms and noticeable changes in laboratory parameters of VOD occurred in these patients at a median on day 18 (range day 13 – 28) after HSCT. All 5 patients had significantly increased serum levels of IL-6 (*P* = 0.0313), IL-8 (*P* = 0.0156) and TNF-α (*P* = 0.0313) compared to the baseline before start of conditioning (Table 2). This occurred at the same time or shortly before (median 2 days, range 1–3 days) clinical symptoms were diagnosed (Table 2). None of the 5 pediatric patients with VOD had a GvHD grade III or IV or a sepsis simultaneously.

**Table 2:** Cytokine levels at baseline before and at the beginning of the first clinical symptoms of veno-occlusive-disease and organ specific graft-versus-host disease

Post-transplant-adverse event	Serum level	Before post-transplant adverse event		Post-transplant adverse event		
		Median	Range	Median	Range	*P*-value
VOD	IL-6 pg/ml	3.150	2.0 – 28.0	69.850	7.7 – 197.0	**0.0313**
	IL-8 pg/ml	18.4	5.0 – 54.2	105	13.6 – 200.0	**0.0156**
	IL-10 pg/ml	3.55	2.4 – 4.7	6.20	4.3 – 20.4	n.d.
	sIL-2R U/ml	1519.0	397 – 3796	3218.0	1934 – 4666	0.3750
	IL-1 ß ng/ml	0.260	0.15 – 0.39	0.335	0.1 – 0.47	0.7500
	TNF-α pg/ml	8.00	7.5 – 21.8	22.30	10.5 – 103	**0.0313**
Acute liver GvHD	IL-6 pg/ml	2.0	2.0 – 2.0	28.1	27.5 – 28.7	n.d.
	IL-8 pg/ml	20	5.0 – 35.0	526.5	460.0 – 593.0	n.d.
	IL-10 pg/ml	2.00	2.0 – 2.0	135.90	98.8 – 173.0	n.d.
	sIL-2R U/ml	823	823 – 823	5705.0	2483 – 8927	n.d.
	TNF-α pg/ml	4.00	4.0 – 4.0	50.35	32.2 – 68.5	n.d.
Acute intestinal GvHD II-IV	IL-6 pg/ml	3.3	2.0 – 26.0	24.3	2.0 – 254.0	**0.0010**
	IL-8 pg/ml	26.65	5.0 – 5702	33.6	10.7 – 460.0	0.2036
	IL-10 pg/ml	3.20	2.0 – 7.9	46.25	12.4 – 173	**0.0039**
	sIL-2R U/ml	823.5	397 – 2393	2321.0	1296 – 3586	**0.0020**
	IL-1 ß ng/ml	0.21	0.22 – 0.41	0.21	0.17 – 0.31	0.5000
	TNF-α pg/ml	7.20	5.2 – 19.90	28.05	12.4 – 90.6	**0.0020**
Acute skin GvHD II-IV	IL-6 pg/ml	2.15	2.0 – 7.4	8.3	2.5 – 83.2	**0.0010**
	IL-8 pg/ml	10.4	5.0 – 40.0	11.9	5.0 – 47.9	0.2783
	IL-10 pg/ml	4.90	2.0 – 7.7	6.60	1.9 – 278.0	0.9453
	sIL-2R U/ml	930.5	397 – 2831	1846.0	918 – 8540	**0.0049**
	IL-1 ß ng/ml	0.18	0.1 – 0.39	0.34	0.23 – 0.36	0.5000
	TNF-α pg/ml	7.20	4.0 – 17.0	18.20	11.2 – 35.7	**0.0020**

*P-value: statistical comparison between baseline measurements and during post-transplant adverse events by the Wilcoxon matched pairs signed rank test; n.d. = not determined due to small sample size

#### Acute GvHD

An acute GvHD appeared in 24 (39.3 %) out of 61 patients. 11 (45.8 %) of these 24 patients experienced a grade I, 9 (37.5 %) a grade II, 3 (12.5 %) a grade III and 1 (4.2 %) a grade IV GvHD (Table 1). 9 (37.5 %) patients developed an isolated acute organ GvHD; 6 (25 %) occurred in the skin, 2 (8.3 %) were isolated intestinal GvHD and one (4.2 %) occurred as an isolated liver GvHD. *Liver GvHD:* Two of the pediatric patients experienced liver GvHD stage III and stage IV, respectively. In both cases, a clear increase in the levels of IL-6, IL-8, IL-10, sIL-2R and TNF-α could be observed. In one patient, these increases occurred two days before laboratory chemical changes were seen for direct and indirect bilirubin and the transaminases ALT and AST. In the other patient, the cytokine levels and the laboratory chemical markers changed simultaneously. However, due to the small number of cases, it was not possible to detect any statistical significance (Table 2). *Intestinal GvHD:* In 9 (14.8 %) of the 61 patients an acute intestinal GvHD was observed. Acute intestinal GvHD stage I occurred in 2 (22.2 %) patients, while intestinal GvHD stage II was seen in 6 (66.7 %) . One patient (11.1 %) suffered from intestinal GvHD stage III. At the onset of the first clinical symptoms of acute intestinal GvHD with an increase of feces quantity, significant increases of IL-6 (*p* = 0.0010), IL-10 (*p* = 0.0039), sIL-2R (*p* = 0.0020), and TNF-α (*P* = 0.0020) were observed in all pediatric patients with acute intestinal GvHD stage II and III. In both patients with intestinal GvHD stage I, there was only an increase in cytokine levels of sIL-2R and IL-10. The cytokine levels of IL-8 and IL-1ß did not significantly change in the 9 patients with intestinal GvHD stage I to III (Table 2). *Skin GvHD:* A total of 15 (24.59 %) of the 61 pediatric patients experienced an acute GvHD of the skin. 6 (40 %) out of 15 patients had a skin GvHD stage I. 8 (53.3 %) patients suffered from skin GvHD stage II, while 1 (6.67 %) patient experienced acute skin GvHD stage III. The 9 patients with a skin GvHD stage II and III, developed significant increases of IL-6 (*P* = 0.0010), sIL-2R (*P* = 0.0049) and TNF-α (*P* = 0.0020) in the serum, whereas IL-8, IL-10 and IL-1ß did not significantly change. 8 of the 9 patients with skin GvHD stage II and III had an increase in IL-6 a few days before (median 2 days) the appearance of exanthema of the skin. Cytokines sIL-2R and TNF-α increased in all 9 pediatric patients with the appearance of exanthema. The patients with skin GvHD stage I had either no changes in cytokine levels or only an increase of IL-6 (Table 2).

#### Sepsis and bacterial infections

11 (18.0 %) of the 61 patients developed sepsis. 5 (8.2 %) patients had a bacteremia with positive blood cultures. In 21 (34.4 %) out of 61 patients 25 localized bacterial infections were detected over the course of the observation period. Bacterial infections appeared in the urine (*n* = 11), feces (*n* = 8), throat (*n* = 5) and bronchoalveolar lavage (*n* = 1). In the case of sepsis a significant increase of IL-6 (*P* = 0.0020), IL-8 (*P* = 0.0020), sIL-2R (*P* = 0.0156), and TNF-α (*P* = 0.0078) was observed in all pediatric patients (*n* = 8) for which an analysis of the cytokine levels was performed on the day of the occurrence of sepsis. These patients also had a body temperature ≥38.3 °C at that point in time. In the remaining 3 patients that experienced a sepsis, the final analysis of cytokine levels was done more than 24 h prior. Fever was not present at the time blood was taken. There was no change in the analyzed cytokine levels at this time. In all 5 (10.42 %) of the 61 patients diagnosed with bacteremia, a significant increase of IL-6 (*P* < 0.0001) and IL-8 (*P* = 0.0006) was observed (Table 3).

**Table 3:** Cytokine levels at baseline before and at the beginning of the first clinical symptoms of post-transplant related infections

Post-transplant-adverse event	Serum level	Before post-transplant adverse event		Post-transplant adverse event		
		Median	Range	Median	Range	*P*-value
Sepsis	IL-6 pg/ml	11.90	2.0 – 73.6	133.00	23.6 – 2856	**0.0020**
	IL-8 pg/ml	40.0	9.0 – 212.0	650.0	73.4 – 10,385	**0.0020**
	IL-10 pg/ml	7.20	3.6 – 55.9	16.70	14.3 – 98.8	0.0625
	sIL-2R U/ml	1780.0	973 – 5933	4899.0	1387 – 9680	**0.0156**
	IL-1 ß ng/ml	0.14	0.1 – 1.99	1.51	0.17 – 3.54	0.0625
	TNF-α pg/ml	5.6	4.0 – 25.7	26.9	6.0 – 204.0	**0.0078**
Bacteremia	IL-6 pg/ml	3.7	2 – 205	13.8	2.5 – 267	**<0.0001**
	IL-8 pg/ml	21.1	7.5 – 220	35.2	7.70 – 954	**0.0006**
	IL-10 pg/ml	4.0	1.1 – 26.2	5.5	1.0 – 62.3	0.059
	sIL-2R U/ml	1306.0	296 – 6690	1423	414 – 6689	0.2023
	IL-1 ß ng/ml	0.1	0.1 – 0.35	0.20	0.10 – 1.08	0.0026
	TNF-α pg/ml	8.9	4.0 – 26.4	10.8	4.0 – 456	0.0007
Viremia	IL-6 pg/ml	9.2	2.0 – 83.2	11.30	2.0 – 113	**0.0008**
	IL-8 pg/ml	16.00	6.7 – 67.10	18.40	5.0 – 295	0.1867
	IL-10 pg/ml	4.10	1.0 – 35.9	4.5	1.0 – 134	0.3884
	sIL-2R U/ml	1561.0	461 – 4666	1409	322 – 7284.0	0.7676
	IL-1 ß ng/ml	0.1	0.1 – 0.87	0.2	0.1 – 1.48	0.1755
	TNF-α pg/ml	11.9	4.0 – 127.0	12.2	4.0 – 137.0	0.2909
Fungemia	IL-6 pg/ml	5.00	2.0 – 16.0	7.75	2.0 – 267.0	0.1272
	IL-8 pg/ml	23.2	5.0 – 99.9	22.35	5.0 – 243.0	0.5688
	IL-10 pg/ml	3.35	1.6 – 5.1	4.90	2.3 – 39.6	0.4375
	sIL-2R U/ml	1180.0	397 – 2365	1037.0	322 – 3195	0.1289
	IL-1 ß ng/ml	0.1	0.1 – 0.43	0.15	0.1 – 1.01	0.5312
	TNF-α pg/ml	10.9	4.0 – 89.1	9.5	4.0 – 61.1	0.2891

*P*-value: statistical comparison between baseline measurements and during post-transplant adverse events by the Wilcoxon matched pairs signed rank test

#### Viral infections

8 (13.2 %) of 61 patients had a viremia (Table 3). CMV invasive infection was observed in the blood of 6 patients and an ADV infection was found in the blood of 5 patients. Consequently, both infections occurred in 3 patients. These patients had CMV and ADV infection in the post-transplant period. In all 11 invasive viral infections there was a significant increase of IL-6 (*P* = 0.0008). These increases occurred at median 3 days (range 1–4 days) prior to positive PCR testing (Table 3).

#### Fungal infections

6 (9.84 %) of the 61 patients experienced fungemia. In one case, a probable invasive fungal infection came about, while 5 cases showed a possible invasive fungal infection. In all 6 cases, there were positive signs of *Aspergillus* galactomannan antigen in the blood in at least two consecutive samples. No proven invasive fungal infections were observed. No specific cytokine pattern and no significant alterations of IL-1ß, sIL-2R, IL-6, IL-8, IL-10 and TNF-α could be observed in any of these cases (Table 3).

### Clinically relevant comparisons between infectious post-transplant adverse events

In order to better distinguish between the different types of infections, a statistical comparison of cytokine levels was made during infectious post-transplant adverse events that are often clinically difficult to distinguish from each other. A comparison of the occurrence of sepsis, bacteremia, viremia and fungemia was done with all of the examined cytokines.

#### IL-1β

**Fig. 1:**
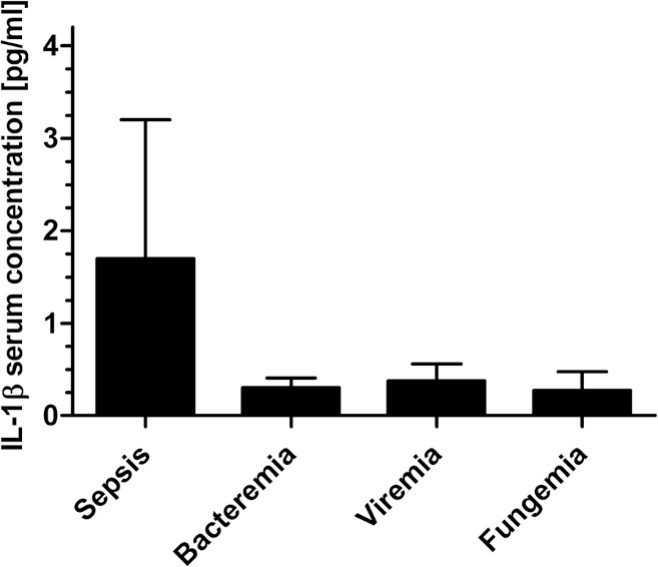
IL-1β concentrations during post-transplant infectious complications. Data show mean IL-1β serum concentrations in occurrence of sepsis (1.7 ± 1.21 pg/ml), bacteremia (0.3 ± 0.27 pg/ml), viremia (0.4 ± 0.39 pg/ml) and fungemia (0.3 ± 0.29 pg/ml). Data show mean +95 % confidence interval (CI)

The comparison of IL-1β values for sepsis (1.7 ± 1.21 pg/ml) and bacteremia (0.3 ± 0.27 pg/ml; *P* = 0.125), sepsis and viremia (0.4 ± 0.39 pg/ml; *P* = 0.062), sepsis and fungemia (0.3 ± 0.29 pg/ml; *P* = 0.125), bacteremia and viremia (*P* = 0.843), bacteremia and fungemia (*P* = 0.625), and viremia and fungemia (*P* = 0.875) showed no significance after Bonferroni correction (adjusted α = 0.0083) (Fig. 1).

#### sIL-2R

**Fig. 2:**
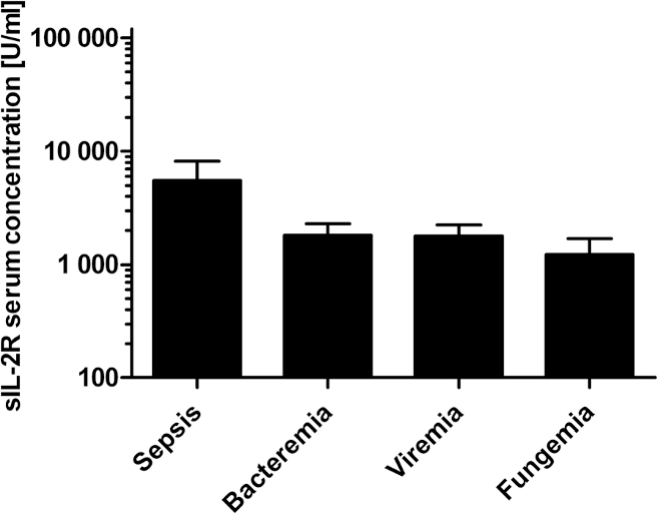
sIL-2R concentrations during post-transplant infectious complications. Data show mean sIL-2R serum concentrations in occurrence of sepsis (5509 ± 2908 U/ml), bacteremia (1803 ± 1416 U/ml), viremia (1783 ± 1374 U/ml) and fungemia (1224 ± 805 U/ml). Data show mean +95 % CI

sIL-2R serum levels during sepsis (5509 ± 2908 U/ml) and bacteremia (1803 ± 1416 U/ml; *P* = 0.250), sepsis and viremia (1783 ± 1374 U/ml; *P* = 0.375), bacteremia and viremia (*P* = 0.983), bacteremia and fungemia (1224 ± 805 U/ml; *P* = 0.431), and viremia and fungemia (*P* = 0.695) were not significantly different. There was a significant difference (*P* = 0.031) between sepsis (5509 ± 2908 U/ml) and fungemia (1224 ± 805 U/ml). However, this was insignificant after Bonferroni correction (adjusted α = 0.0083) (Fig. 2).

#### IL-6

**Fig. 3:**
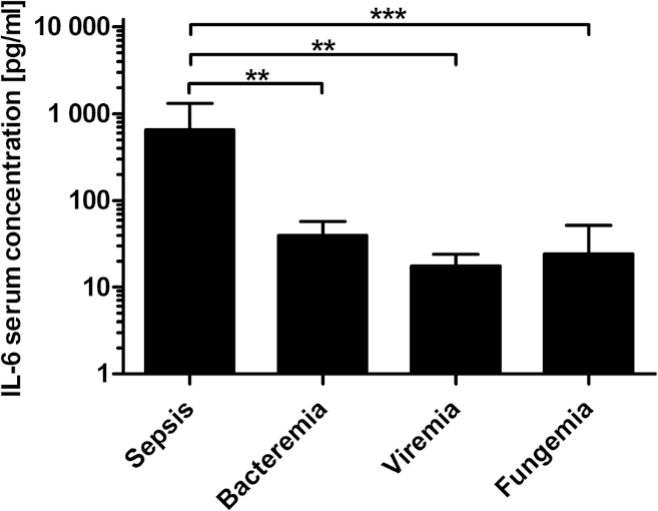
IL-6 concentrations during post-transplant infectious complications. Data show mean IL-6 serum concentrations in occurrence of sepsis (650 ± 989 pg/ml), bacteremia (39.63 ± 58.58 pg/ml), viremia (17.47 ± 22.40 pg/ml) and fungemia (24.07 ± 58.85 pg/ml). Data show mean +95 % CI; **: *P* < 0.01; ***: *P* < 0.001

A significant decrease (*P* = 0.002) of IL-6 was found between sepsis (650 ± 989 pg/ml) and bacteremia (39.63 ± 58.58 pg/ml), as well as between sepsis and viremia (17.47 ± 22.40 pg/ml; *P* = 0.002). There was also a significant difference (*P* = 0.001) between sepsis and fungemia (24.07 ± 58.85 pg/ml). After Bonferroni correction (adjusted α = 0.0083) the decrease of IL-6 between bacteremia and viremia (*P* = 0.0094) and between bacteremia and fungemia, (*P* = 0.0194) was not significant. The comparison of serum IL-6 concentration between viremia and fungemia (*P* = 0.305) was neither significant (Fig. 3).

#### IL-8

**Fig. 4:**
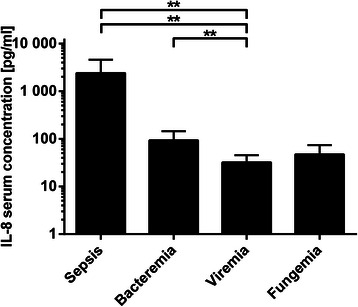
IL-8 concentrations during post-transplant infectious complications. Data show mean IL-8 serum concentrations in occurrence of sepsis (2406 ± 3190 pg/ml), bacteremia (93.53 ± 168.80 pg/ml), viremia (32.31 ± 46.23 pg/ml) and fungemia (47.26 ± 56.93 pg/ml). Data show mean +95 % CI; **: *P* < 0.01

There was a significant decrease (*P* = 0.002) of IL-8 between sepsis (2406 ± 3190 pg/ml) and bacteremia (93.53 ± 168.8 pg/ml), as well as between sepsis and viremia (32.31 ± 46.23 pg/ml; *P* = 0.002), and between bacteremia and viremia (*P* = 0.0064). There was a decrease (*P* = 0.0341) between sepsis and fungemia (47.26 ± 56.93 pg/ml). This was, however, insignificant after Bonferroni correction. The comparison of IL-8 serum concentration between viremia and fungemia (*P* = 0.161) and between bacteremia and fungemia (*P* = 0.15), showed no significant changes. Due to the large scattering of the measured values and high maximum values, the standard deviations are often much larger than the mean values (Fig. 4).

#### IL-10

**Fig. 5:**
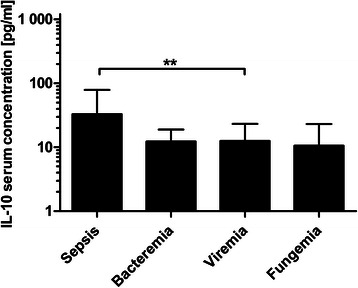
IL-10 concentrations during post-transplant infectious complications. Data show mean IL-10 serum concentrations in occurrence of sepsis (32.82 ± 36.91 pg/ml), bacteremia (12.28 ± 15.96 pg/ml), viremia (12.60 ± 26.47 pg/ml) and fungemia (10.59 ± 13.39 pg/ml). Data show mean +95 % CI; **: *P* < 0.01

A significant decrease (*P* = 0.005) of IL-10 serum concentration was found between sepsis (32.82 ± 36.91 pg/ml) and viremia (12.60 ± 26.47 pg/ml). All other pairwise comparisons were statistically insignificant. IL-10 serum concentrations in occurrence of sepsis showed no significant changes in comparison to bacteremia (28.12 ± 15.96 pg/ml). The comparison of sepsis with the fungemia (10.59 ± 13.39 pg/ml), also showed no significant change. When comparing viremia and fungemia (*P* = 0.50), bacteremia and fungemia (*P* = 0.50) and bacteremia and viremia (*P* = 0.31), no significant changes in IL-10 serum concentration were found after Bonferroni correction (adjusted α = 0.0083) (Fig. 5).

#### TNF-α

**Fig. 6:**
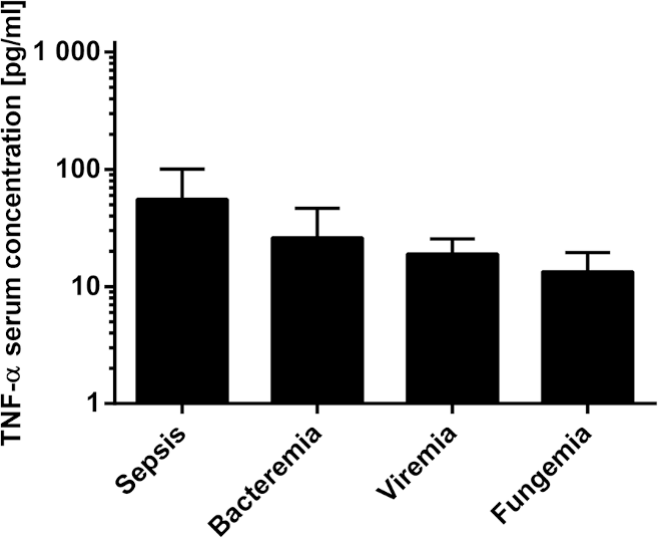
TNF-α concentrations during post-transplant infectious complications. Data show mean TNF-α serum concentration in occurrence of sepsis (55.42 ± 63.04 pg/ml), bacteremia (25.95 ± 68.32 pg/ml), viremia (18.88 ± 22.64 pg/ml) and fungemia (13.37 ± 12.60 pg/ml). Data show mean +95 % CI

After Bonferroni correction, there was no significant decrease (*P* = 0.046) of TNF-α between sepsis (55.42 ± 63.04 pg/ml) and fungemia (13.37 ± 12.60 pg/ml). Sepsis compared to bacteremia (25.95 ± 68.32; *P* = 0.85) and to viremia (18.88 ± 22.64; *P* = 0.322) showed no significant changes in TNF-α serum concentrations. Moreover, the comparison of TNF-α concentrations in occurrence of bacteremia in relation to both fungemia (*P* = 0.614) and viremia (*P* = 0.884) was not significant. A comparison of the TNF-α concentration between viremia and fungemia also showed no significance (*P* = 0.091) after Bonferroni correction (adjusted α = 0.0083) (Fig. 6).

## Discussion

The primary objective of this retrospective investigation was to analyze whether early identification of major post-transplant adverse events in pediatric patients with hemato-oncological malignancies and non-malignancies after HSCT is possible through the examination of cytokine levels. Early identification of these post-transplant adverse events is required for timely and adequate treatment and thus has decisive impact on patient outcome.

This analysis focused on finding markers that could help to differentiate post-transplant complications with similar initial clinical symptoms and laboratory parameters. These include for example, distinguishing a VOD from liver GvHD, a localized viral infection in feces from an intestinal GvHD, and the various types of invasive infections like bacteremia, viremia, and fungemia. In order to do this, we carried out a single center survey that analyzed the cytokine levels of IL-1ß, sIL-2R, IL-6, IL-8, IL-10, sIL-2R and TNF-α during conditioning, and during the post-transplant period in 61 pediatric patients and young adults after allogeneic and autologous HSCT. Occurrences of the first laboratory chemical changes or clinical symptoms of a VOD coincided with a significant increase in cytokine levels of IL-6, IL-8 and TNF-α. This observation is in line with the results of a study in which the levels of TNF-α, IL-6, and IL-8 were analyzed in 53 patients undergoing HSCT. Elevation of these cytokines in association with hepatic dysfunction (defined as increased bilirubin levels) also occurred in VOD patients [34]. In another analysis of adult transplanted patients high IL-8 levels were detected during severe VOD in 6 patients, 5 of whom showed elevated levels of IL-6 [13]. Furthermore, a study of 10 patients with VOD found a significant increase in the levels of sIL-2R (*P* < 0.001) with mean values of 4546 ± 1420 U/ml in contrast with a control group without major complications after HSCT [14]. When this is compared to the values observed in the present trial, which displayed a median of 3218 U/ml, similarly significant values can be observed (*P* = 0.0011) when compared to the levels detected in the healthy control group (median 585 U/ml, range 296.0–869.0 U/ml). In cases of liver GvHD, there was a further increase in sIL-2R and IL-10 along with the increase in levels of cytokine IL-6, IL-8, and TNF-α. These two markers, sIL-2R and IL-10, may be used to differentiate a VOD from a liver GvHD.

In cases of an intestinal GvHD, the same cytokine pattern was shown as in acute skin GvHD, with a significant increase in sIL-2R, IL-6 and TNF-α. The only differentiator of intestinal GvHD was the additional significant increase of IL-10 in comparison to the skin GvHD, which showed no changes in IL-10 levels. Several studies have shown that elevated TNF-α levels after HSCT are associated with the presence of acute GvHD and that the TNF-α levels increase nearly simultaneously with the onset of acute GvHD [17, 20, 35]. The limited published data on cytokine levels of relevant viral infections in immunosuppressed patients show similar results. In an analysis of 14 patients with a reactivation of HHV-6, IL-6 levels were significantly higher than in patients without HHV-6 activation [9].

However, in the present analysis it was difficult to differentiate a viremia from a bacteremia by examining cytokine levels. In both bacteremia and viremia, an isolated significant increase of IL-6 was observed. In another retrospective study, significant increases of IL-6, IL-8 and sIL-2R were observed during the analysis of febrile episodes before bacteremia caused by gram-negative bacteria [36]. In pediatric patients with sepsis, a cytokine storm occurred with an increase of sIL-2R, IL-6, IL-8, and TNF-α [37]. The observations of the present investigation are also consistent with another analysis of 79 pediatric patients with sepsis. These patients displayed significantly higher TNF-α levels than patients with negative blood cultures [38].

In the present analysis, the cytokines IL-6, IL-8 and IL-10 played a central role when differentiating between the different types of infectious post-transplant complications. It could be found that the cytokine IL-6 can significantly distinguish between sepsis and fungemia (*P* = 0.0010), sepsis and viremia (*P* = 0.0020), and sepsis and bacteremia (*P* = 0.0020). However, a differentiation between bacteremia and viremia, bacteremia and fungemia, and viremia and fungemia was not significant. Furthermore, the cytokine IL-8 enabled significant differentiation (*P* = 0.0020) between sepsis and viremia and sepsis and bacteremia. A distinction between sepsis and fungemia was not possible. In addition, IL-8 facilitates a significant distinction between bacteremia and viremia (*P* = 0.0064), and IL-10 can differentiate between sepsis and viremia (*P* = 0.005).

## Conclusions

The presented retrospective survey shows that the analysis of cytokines enables differentiation of major post-transplant complications. A significant increase in cytokine levels of IL-6, IL-8, and TNF-α announces the beginning of a VOD. For suspected cases of intestinal GvHD ≥ grade II, a significant increase of cytokines IL-6, IL-10, sIL-2R and TNF-α may serve as an early identification marker. A significant increase of IL-6 alone was associated with ADV-viremia and significant increases of IL-6 and IL-8 with bacteremia. Separate from this, a sepsis was characterized by significant increases of IL-6, IL-8 and sIL-2R. Analysis of the cytokines allowed differentiation of post-transplant adverse events with similar clinical symptoms (for example intestinal GvHD and diarrhea due to viral infection, or VOD and liver GvHD). However, studies with larger patient cohorts and a prospective setting will be performed to validate these conclusions in order to use characteristic cytokine patterns to identify post-transplant adverse events as early as the onset of fever with unknown origin or other initial clinical symptoms, and thus facilitate a correct treatment approach.

## Abbreviations

ADV: Adenovirus
ATG: Anti-thymocyte globulin
CMV: Cytomegalovirus
CsA: Cyclosporine A
EBV: Epstein-Barr virus
ELISA: Enzyme linked immunosorbent assay
EORTC: Invasive Fungal Infections Cooperative Group of the European Organization for Research and Treatment of Cancer
G-CSF: Granulocyte colony-stimulating factor
GvHD: Graft-versus-Host disease
HHV-6: Human herpes virus 6
HSCT: Hematopoietic stem cell transplantation
HSV: Human herpes simplex virus
IL-10: Interleukin 10
IL-1β: Interleukin 1β
IL-6: Interleukin 6
IL-8: Interleukin 8
mg/kg BW/d: Milligram per kg body weight per day
MMFD: Mismatched family donor
MSG: National Institute of Allergy and Infectious Diseases Mycoses Study Group
MUD: Matched unrelated donor
SD: Standard deviation
sIL-2R: Soluble interleukin-2 receptor
TNF-α: Tumor necrosis factor-α
VOD: Veno-occlusive disease
VZV: Varicella zoster virus
